# Does One Size Really Fit All? Morphometric Analysis of Distal Femur and Proximal Tibia in an Ethnic Indian Population and Correlation to the Sizing of Existing Total Knee Arthroplasty Implants

**DOI:** 10.7759/cureus.30824

**Published:** 2022-10-29

**Authors:** Mithilesh Ranjan, Namith Rangaswamy, Siva Srivastava Garika, Chandrashekhar Yadav

**Affiliations:** 1 Department of Orthopedics, All India Institute of Medical Sciences, New Delhi, New Delhi, IND; 2 Department of Orthopedics, Primus Super Speciality Hospital, New Delhi, New Delhi, IND

**Keywords:** proximal tibia, distal femur, implant sizes, osteoarthritis, total knee arthroplasty implants, morphometric analysis, total knee arthroplasty

## Abstract

Background: Total knee arthroplasty (TKA) has been proven to be a highly efficacious procedure for patients with end-stage osteoarthritis who have persistent symptoms not managed by conservative treatment. A large percentage of standard commercially available total knee arthroplasty (TKA) implants are imported and designed based on morphometric data of Western populations, which are known to have a larger build compared to their Asian counterparts. Hence, these prostheses may sometimes not be the best fit for Indian patients. We conducted this study to examine the anthropometry of osteoarthritic knees of Indian patients, analyze anatomical differences between males and females, and compare these measurements with commercially available five TKA implants.

Methods: Morphometric data were collected from 150 Indian patients with osteoarthritis of the knee using computed tomography (CT) scans. The mediolateral (ML) and anteroposterior (AP) dimensions of the distal femur and proximal tibia were measured, and aspect ratios (ML/AP) were calculated. These measurements were correlated with current commercially available implant sizes.

Results: We examined CT scans of 100 female and 50 male patients' knees with a combined average age of 58.2 ± 7.5 years. The mean mediolateral and anteroposterior dimensions of the distal femur for Indian knees were 74.5 ± 5.8 mm and 58.0 ± 4.2 mm, respectively, whereas for the proximal tibia, 69.1 ± 5.5 mm and 43.8 ± 3.6, respectively. The mean aspect ratio for the femur was 129.0 ± 6.0 and for the tibia was 158.1 ± 9.1. Male dimensions were found to be greater than female dimensions in all measured aspects of the distal femur and proximal tibia for the Indian population. However, the aspect ratio of the tibia was not found to vary with gender. When compared with the dimensions of other ethnic groups, the size of Indian knees was found to be smaller than Caucasians.

Conclusions: There is a mismatch between the anatomy of Indian knees and currently available TKA implants, and these implants may have drawbacks when implanted in Indian patients. The obtained anthropometric data may provide useful directions for designing TKA implants of more suitable sizes and aspect ratios for Indian patients.

## Introduction

Osteoarthritis is the most common degenerative joint disease not only in India but in the world as well. The overall prevalence of osteoarthritis in India is estimated to be 28.7% [1]. With advancing healthcare, total knee arthroplasty (TKA) is established as a standard management strategy to treat severe osteoarthritis, relieve persistent knee pain, provide better knee function, and improve the patient’s quality of life [1]. The long-term success of the TKA procedure depends largely on accurate bone cuts, adequate soft tissue balancing, and prosthesis selection that appropriately matches the dimensions of the cut surfaces of the distal femur and proximal tibia [2,3]. Complications may arise due to improper sizing and mismatch of implants. The overhang of prostheses can result in soft tissue irritation and impingement, whereas under-sizing can lead to subsidence and instability [4,5]. Various TKA implants are commercially available with unique shapes and designs. Most of the commonly available TKA implants are designed based on anthropometric data from the Western population, which are known to have a larger build and structure than their Asian counterparts [4,6-10]. This discrepancy in the reference data for implant designs can lead to a mismatch between the TKA component and the cut bony surfaces upon usage in ethnic Asian patients. We conducted this computed tomography (CT) scan-based study to determine the disparity between the commercially available TKA implant sizing and the anthropometric measurements in the Indian population and to provide reference data along with recommendations for implant sizes to fit the Indian population. To our knowledge, this is the largest morphometric study done to date on the ethnic Indian population.

## Materials and methods

In this cross-sectional observational study, we performed CT scans on consecutive 150 Indian ethnic volunteer patients (150 knees) with osteoarthritis presenting to our outpatient clinic, a tertiary center in North India. The institutional ethical board clearance was obtained before the commencement of the study and written informed consent was obtained from all participants prior to enrolment. Patients who had undergone any previous surgery on the knee, patients having Charcot's joint, rheumatoid arthritis, and ankylosing arthritis were excluded from the study. Patients with concomitant hip or spine deformities, patients with a congenital deformity of the lower limb, flexion deformity of more than 10 degrees at the knee, patients with pathological fractures and tumors around the knee joint, and patients with a history of knee injury within the last six months were also excluded. All subjects underwent a computed tomography scan of the knee joint using a multi-detector (MD) CT scanner (SOMATOM Sensation 40, Siemens, Germany). During the scan, the subjects were positioned supine with their lower limbs extended and the patella pointing upward. This position was sustained throughout the scan to standardize the procedure and minimize any fallacy in measurements. The CT scan was acquired with a slice thickness of 0.6 mm for heightened accuracy. All images were acquired in digital imaging and communications in medicine (DICOM) document format and exported to a dedicated workstation, Syngo (Siemens, Germany), to measure the morphologic parameters.

**Figure 1:**
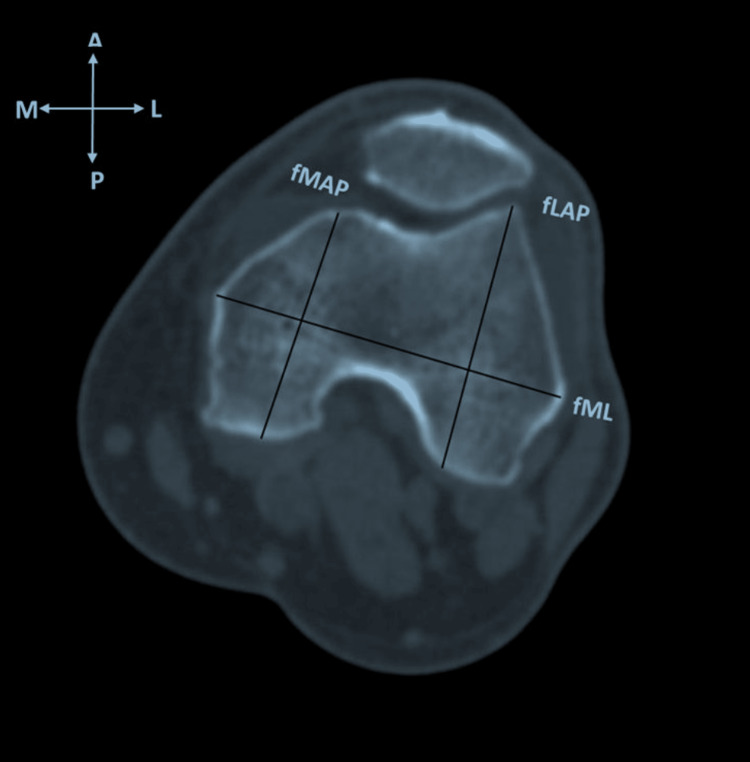
Axial CT image of the distal femur showing mediolateral length (fML), lateral anteroposterior length (fLAP), and medial anteroposterior length (fMAP). CT: computed tomography; fML: distal femoral mediolateral length; fLAP: distal femoral lateral anteroposterior length; fMAP: distal femoral medial anteroposterior length.

In the distal femur, an axial image perpendicular to the transepicondylar line in the frontal plane, at a distance of 9 mm proximal to the joint line was studied. This level generally corresponds to the level of resection of the distal femur in TKA. The following measurements were taken: (i) distal femoral mediolateral length (fML): the extent of the line connecting the most prominent points over the medial and lateral condyles. (ii) Medial condyle anteroposterior length (fMAP): extent of the line drawn perpendicular to the fML line and passing across a most posterior point in the medial condyle. (iii) Lateral condyle anteroposterior length (fLAP): the length of a line drawn perpendicular to the fML line and passing across the most posterior point in the lateral condyle (Figure 1).

(iv) Distal femoral anteroposterior length (fAP): denoted as a maximum of the medial and lateral anteroposterior lengths (fMAP and fLAP). (v) Femoral aspect ratio (fR): calculated as a mediolateral dimension (fML) divided by anteroposterior dimension (fAP) × 100.

**Figure 2:**
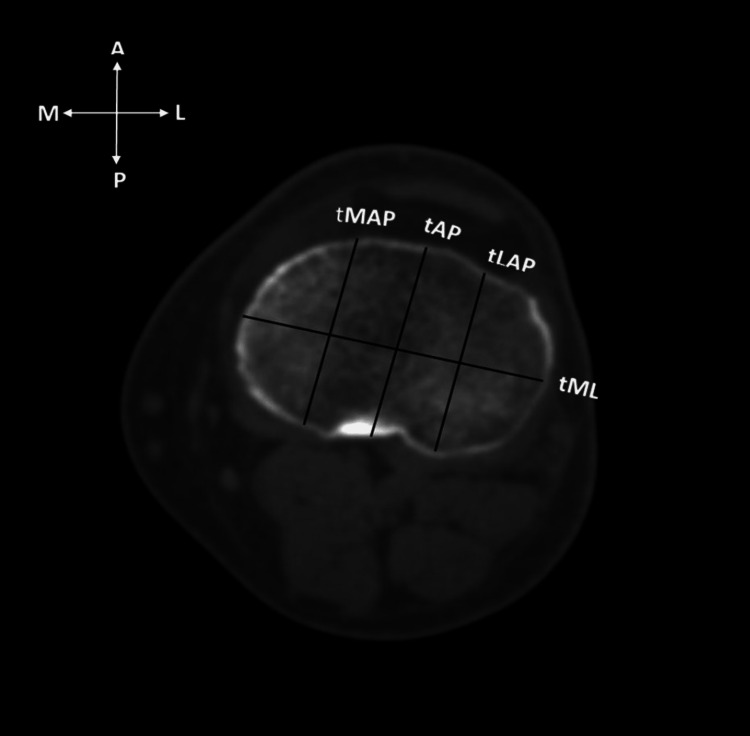
Axial CT image of the proximal tibia showing mediolateral length (tML), lateral anteroposterior length (tLAP), anteroposterior length (tAP), and medial anteroposterior length (tMAP). CT: computed tomography; tML: proximal tibial mediolateral length: tLAP: lateral anteroposterior length; tAP: anteroposterior length; tMAP: medial anteroposterior length.

In the proximal tibia, an axial image at the right angle to the longitudinal axis of the tibia, at the level of maximum mediolateral length, was taken for measurements. The following measurements were taken: (i) mediolateral length of the tibia (tML): measured as the span of the longest horizontal line in axial images of the proximal tibia parallel to the transepicondylar axis of the femur, (ii) medial anteroposterior length (tMAP): measured as the span of the line drawn parallel to tAP and passing through the posterior-most point of the medial tibial condyle, (iii) anteroposterior length of the tibia (tAP): the extent of a line drawn perpendicular and passing across the midpoint of the tibial mediolateral line (tML), (iv) lateral anteroposterior length (tLAP): length of a line drawn parallel to tAP and passing right across the posterior-most point of the lateral tibial condyle, and (v) tibial aspect ratio (tR) calculated as the ratio of mediolateral dimension to anteroposterior dimension multiplied by 100 (Figure 2).

The morphometric data obtained were analyzed for gender differences and compared with five commercially available TKA prosthetic systems currently in India: NexGen® (Zimmer Biomet, Warsaw, Indiana), Scorpio NRG® (Stryker Co., Mahwah, New Jersey), SIGMA® (DePuy Orthopedics, Inc, Warsaw, Indiana), Triathlon® (Stryker Co., Mahwah, New Jersey), and Freedom® (Meril, Vapi, Gujarat). In the statistical analysis, data were collected and managed in Microsoft Excel (Microsoft Corporation, California). Analysis was done using IBM® SPSS® Software (IBM SPSS Statistics for Windows, Version 28.0., IBM Corp., Armonk, NY). Descriptive statistics were used for demographic data and CT scan measurements. T-test was used for comparative analysis of continuous data between genders. Best fit lines were calculated with the use of the least square regression method. A p-value of <0.01 was considered a statistically significant outcome.

## Results

Our study consisted of 100 female and 50 male patients with a combined average age of 58.2 ± 7.5 years. Table 1 summarizes the demographic data of our study subjects. In our study, there was no statistical difference between height, age, weight, or side (72 left and 78 right) of the examined knee.

**Table 1:** Summary of demographic data. cm: centimeter; kg: kilogram; kg/m^2^: kilogram per square meter.

	Males (n = 50)	Females (n = 100)	Combined (n = 150)
Age (years)	59.7 ± 7.2	57.4 ± 7.5	58.2 ± 7.5
Height (cm)	167.7 ± 6.8	153.8 ± 7.4	158.4 ± 9.7
Weight (kg)	73.6 ± 11.4	69.1 ± 11.4	70.6 ± 11.5
Body mass index (BMI) (kg/m^2^)	26.2 ± 3.4	29.2 ± 4.6	28.2 ± 4.5

In the distal femur morphology, the average anteroposterior (fAP) and mediolateral (fML) dimensions for the Indian population were 58.0 ± 4.2 mm and 74.5 ± 5.8 mm, respectively. The mean fML dimensions in males and females were 81.0 ± 3.8 mm and 71.3 ± 3.5 mm, respectively. The average fAP in males was 61.9 ± 3.1, whereas in females it was 56.0 ± 3.1. The mean measurement values of fMAP and fLAP dimensions of the Indian population were 54.6 ± 4.3 mm and 57.8 ± 4.2 mm, respectively. Males had significantly larger values of fML, fAP, fMAP, and fLAP dimensions when compared with females (p < 0.0001). Also, males were found to have a statistically significantly larger femoral aspect ratio (fR) than females (p = 0.0006) (Table 2).

**Table 2:** Distal femur morphologic measurements in mm. fML: distal femoral mediolateral length; fAP: distal femoral anteroposterior length; fMAP: distal femoral medial anteroposterior length; fLAP: distal femoral lateral anteroposterior length; fR: femoral aspect ratio. *The p-values are calculated for comparison between males and females.

	Combined	Males	Females	*P-value
fML	74.5 ± 5.8	81.0 ± 3.8	71.3 ± 3.5	<0.0001
fAP	58.0 ± 4.2	61.9 ± 3.1	56.0 ± 3.1	<0.0001
fMAP	54.6 ± 4.3	58.3 ± 3.5	52.8 ± 3.4	<0.0001
fLAP	57.8 ± 4.2	61.7 ± 3.2	55.9 ± 3.2	<0.0001
fR (%)	129.0 ± 6.0	131.3 ± 5.5	127.8 ± 6.0	0.0006

In the proximal tibial morphology, the average mediolateral (tML) and anteroposterior (tAP) dimensions for the Indian population were 69.1 ± 5.5 mm and 43.8 ± 3.6 mm, respectively, and the average tMAP and tLAP dimensions of the Indian population were 46.0 ± 4.2 mm and 42.9 ± 4.0 mm, respectively. When compared with females, males had larger values for tML, tAP, tMAP, and tLAP dimensions (p < 0.0997) (Table 3).

**Table 3:** Proximal tibial morphologic measurements in mm. tML: proximal tibial mediolateral length; tAP: proximal tibial anteroposterior length; tMAP: proximal tibial medial anteroposterior length; tLAP: proximal tibial lateral anteroposterior length; tR: tibial aspect ratio. *The p-values are calculated for comparison between males and females.

	Combined	Males	Females	*P-value
tML	69.1 ± 5.5	75.2 ± 3.6	66.1 ± 3.2	<0.0001
tAP	43.8 ± 3.6	47.2 ± 3.0	42.1 ± 2.7	<0.0001
tMAP	46.0 ± 4.2	50.0 ± 3.1	43.9 ± 3.0	<0.0001
tLAP	42.9 ± 4.0	46.9 ± 2.6	40.9 ± 2.9	<0.0001
tR (%)	158.1 ± 9.1	159.8 ± 10.0	157.2 ± 8.6	0.0997

We found a linear correlation between the tML and tAP of both male and female subjects. The tML dimension demonstrated a progressive increase with increasing tAP dimension in both males and females. The tML dimension depicted a progressive increase with increasing tAP dimension in both males and females.

**Figure 3:**
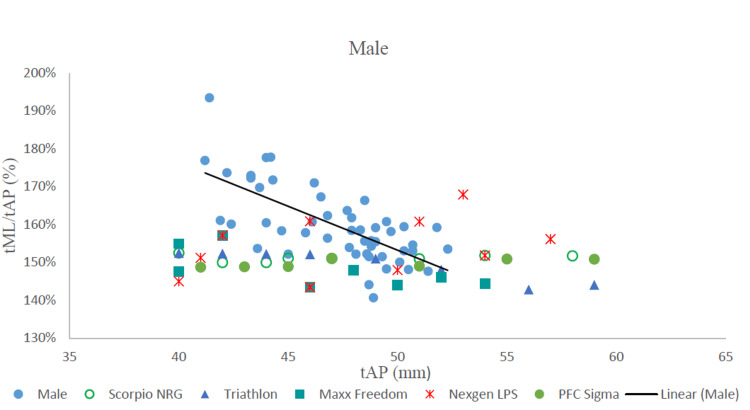
The tibial aspect ratio vs tAP in males and comparison with five implants. tML: tibial mediolateral length; tAP: tibial anteroposterior length; tML/tAP: tibial aspect ratio; mm: millimeters.

**Figure 4:**
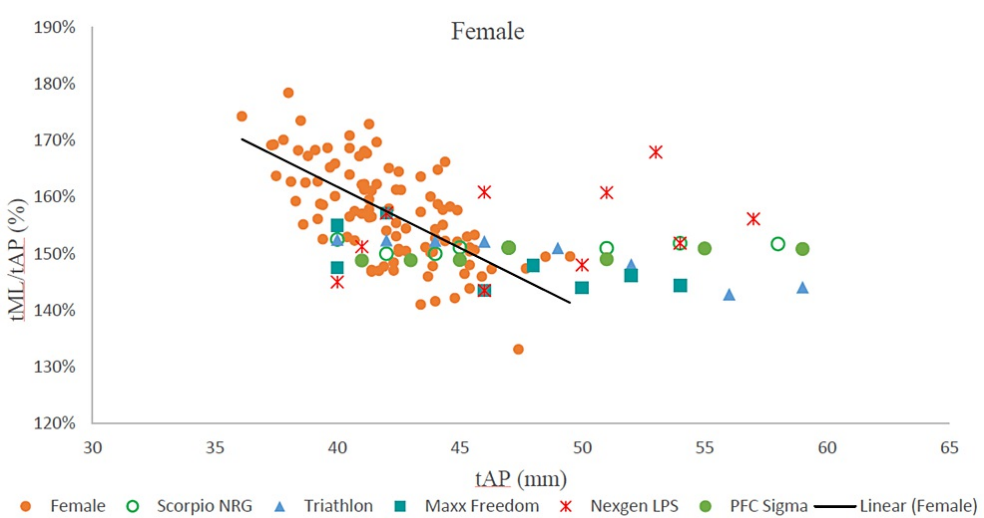
The tibial aspect ratio vs tAP in females and comparison with five implants. tML: tibial mediolateral length; tAP: tibial anteroposterior length; tML/tAP: tibial aspect ratio; mm: millimeters.

On comparing the tibial aspect ratio to the anteroposterior dimension of morphologic data with similar dimensions of commercially available tibial components, we found that none of the five design sizes corresponded to the trend line (Figures 3, 4).

**Figure 5:**
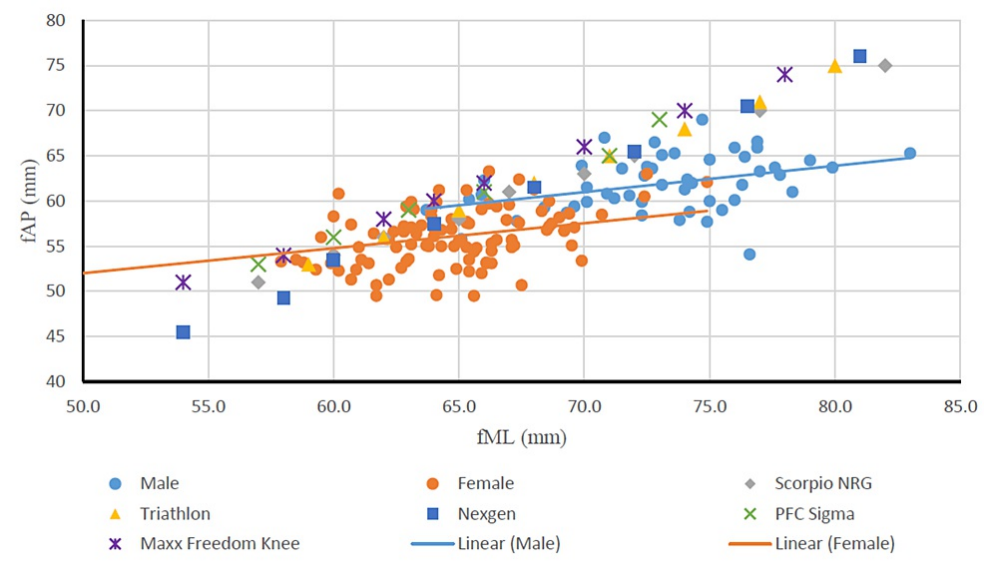
Relationship between fAP and fML of males and females and comparison with femoral components. fAP: distal femoral anteroposterior length; fML: distal femoral mediolateral length; mm: millimeters.

On evaluating the mediolateral length of the distal femur at the level corresponding to the section of the distal femoral bone cut, it was evidently noted that the mediolateral measurements of femoral implants fell short of the population measurements (Figure 5).

**Figure 6:**
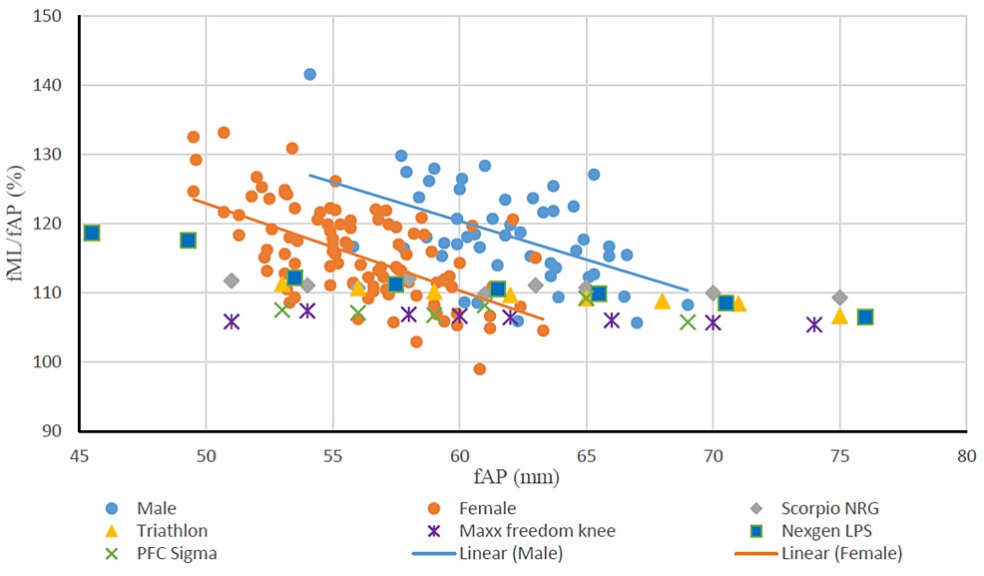
Relationship between fAP and aspect ratio of the femur of males and females and comparison with femoral components. fAP: distal femoral anteroposterior length; fML: distal femoral mediolateral length; fML/fAP: femoral aspect ratio; mm: millimeters.

Upon comparing the fML and fAP of the distal femur of males and females with similar dimensions of femoral implants, the aspect ratio (fR) of the Indian population showed a declining trend with increasing fAP, whereas a consistent aspect ratio was seen in most of the implants with the increase in fAP. None of the implants perfectly matched the dimensions of the study population of either gender (Figure 6).

## Discussion

Total knee arthroplasty (TKA) is considered one of the most successful surgical procedures in orthopedic history. Although there have been continuous development and improvement in designs for TKA prostheses, the functional outcome of the surgery depends on various factors. Baker et al. studied patient satisfaction following total knee arthroplasty utilizing a postal questionnaire sent to 10,000 patients who underwent surgery in England and Wales and found that approximately 18% of patients were "dissatisfied" with their surgical results at one year following their TKA [11]. Any mismatch between the implant and resected bony surfaces may contribute to suboptimal patient satisfaction and functional impairment. Hence, the long-term successful outcome of TKA prostheses depends on appropriate surgical technique as well as a near-perfect match between the resected surface of the knee and the prosthesis [2,3].

Various studies have reported the dimensions and sizes of the human knees through plain radiograph measurements, cadaveric dissections, intraoperative measurements, CT scans, or magnetic resonance imaging (MRI). Nearly all studies done on the Asian population agree that there is a definite difference in morphologic measurements and the commercially available TKA implants and called for the need for a sex-specific prosthesis to better match the male and female knee joint geometry [6-9,12-15]. Morphometrical data and differences among the different ethnic populations are summarized in Tables 4, 5.

**Table 4:** Summary of the morphometry of the distal femur reported by different authors for different ethnic populations. CT: computed tomography, MRI: magnetic resonance imaging; M: male; F: female: 3D: three-dimensional; fML: femoral mediolateral length; fAP: femoral anteroposterior length; fR: femoral aspect ratio; mm: millimeters.

Author	Ethnicity	Sample size		Technique	fML (mm)		fAP (mm)		fR (%)	
		M	F		M	F	M	F	M	F
Yue et al. [12]	Chinese	20	20	CT imaging	82.6 ± 3.6	72.8 ± 2.6	65.0 ± 2.8	58.8 ± 2.5	127 ± 3	124 ± 4
Lim et al. [13]	Koreans	56	59	MRI imaging	81.5 ± 5.1	76.7 ± 3.7	62.7 ± 4.1	58.4 ± 3.1	-	-
Ishimaru et al. [16]	Japanese	40	40	CT 3D reconstruction	82.6 ± 5.8	73.4 ± 3.6	63.4 ± 2.9	58.9 ± 3.6	131 ± 6	125 ± 9
Berger et al. [17]	Caucasians (Americans)	20	15	Cadaver study	85.6 ± 5.1	75.4 ± 2.3	68.1 ± 4.6	60.2 ± 2.0	-	-
Our study	Indian	50	100	CT imaging	81.0 ± 3.8	71.3 ± 3.5	61.9 ± 3.1	56.0 ± 3.1	131.3 ± 5.5	127.8 ± 6.0

**Table 5:** Summary of the morphometry of the proximal tibia reported by different authors for different ethnic populations. CT: computed tomography; M: male; F: female: 3D: three-dimensional; tML: tibial mediolateral length; tAP: tibial anteroposterior length; tMAP: tibial medial anteroposterior length; tLAP: tibial lateral anteroposterior length; tR: tibial aspect ratio; mm: millimeters.

Authors	Ethnicity	Sample size		Technique	tML (mm)		tAP (mm)		tMAP (mm)		tLAP (mm)		tR (%)	
		M	F		M	F	M	F	M	F	M	F	M	F
Cheng et al. [7]	Chinese	94	78	CT 3D reconstruction	76.4 ± 2.8	68.8 ± 4.6	51.3 ± 2.0	45.7 ± 1.9	53.3 ± 2.5	47.5 ± 2.4	47.7 ± 2.7	42.4 ± 2.3	149.0 ± 5.7	150.7 ± 6.1
Kwak et al. [14]	Koreans	50	50	CT 3D reconstruction	76.1 ± 4.0	67.6 ± 3.1	48.2 ± 3.3	43.2 ± 2.3	48.5 ± 3.7	43.5 ± 2.9	43.5 ± 2.9	39.8 ± 2.5	158	156
Uehara et al. [9]	Japanese	21	59	CT imaging	83.0 ± 6.2	71.7 ± 4.0	53.8 ± 6.6	46.6 ± 3.6	-	-	-	-	-	-
Mensch et al. [18]	Caucasians (Americans)	14	16	Cadaveric study	80.3 ± 3.7	46.6 ± 3.6	-	-	54.3 ± 3.6	46.0 ± 2.1	43.5 ± 2.8	38.3 ± 2.6	-	-
Our study	Indian	50	100	CT imaging	75.2 ± 3.6	66.1 ± 3.2	47.2 ± 3.0	42.1 ± 2.7	50.0 ± 3.1	43.9 ± 3.0	46.9 ± 2.6	40.9 ± 2.9	159.8 ± 10.0	157.2 ± 8.6

Iorio et al. [19] compared the long-term results of the posterior cruciate-retaining knee between the American and Japanese cohorts. They found a significantly limited postoperative range of motion when compared with the Western population. Also, they observed that nearly 4.1% of Japanese patients needed revision of TKA within a mean follow-up of 6.6 years, while American patients had a revision rate of only 2.6% at a mean follow-up of nine years. The authors believed that the morphological differences between the different ethnic populations and the fact that the knee implant design was largely based on Western data may have played a role in the differences in outcomes found in their study.

Two decades ago, Vaidya et al. [6] highlighted the issue of disparity among the implant sizes in their study of 86 knees. They pointed out that the mediolateral splaying of the distal femur in the Indian population led to the undersizing of the femoral implants when the implants corresponding to respective anteroposterior measurements were used, especially in females. They made an effort to recommend the femoral component sizes from extra small to large. They hoped for collaborative large-scale manufacturing of smaller knee implants to better fit the Asian population. However, nothing much seems to have changed since. None of the commercially available femoral components of TKAs fell on the morphometric trendline. The tibial aspect ratios of the available implants in our study did not correspond to the Indian proximal tibial aspect ratios. The appropriate mediolateral coverage of the resected distal femoral bone allows uniform stress distribution, adequate tracking of the patella, and easy wound closure. Thilak et al. [20] in their morphological study also found that the femoral component was undersized mediolaterally when used for the corresponding AP dimensions. However, there are little or no studies done to determine the long-term outcome of mediolateral size mismatch in TKAs.

Shah et al. [21] in their study observed that along with the femoral component mismatch, the commercially available implants that have a smaller anteroposterior diameter of the tibia were undersized across the mediolateral dimension, and those with larger anteroposterior measurements showed overhang in the mediolateral dimension.

The life span of a common Indian was 41.7 years in 1960, whereas longevity has leaped to 67.5 and 69.8 years in men and women, respectively, by the year 2020 [22,23]. This increasing longevity has led to an increase in the spectrum of senile orthopedic disorders, including osteoarthritis. With the world’s second-largest population, the problem statement of severe osteoarthritis in India is astronomical, and so is the need for joint replacement. With a large volume of TKAs performed every day, it is pertinent that such implants should be specifically made to fit Indian ethnic morphologies. We urge researchers from similar Asian ethnic countries with smaller built populations to perform collaborative studies and come up with a consensus to recommend the standard sizes for the TKA implants for a better outcome in the long term and also to the industries to consort with such researchers to obtain the reference data to manufacture the implants on a mass scale to better match the Asian population.

## Conclusions

There is a mismatch between the anatomy of Indian knees and currently available TKA implants, and these implants may have drawbacks when implanted in Indian patients. The obtained anthropometric data may provide useful directions for designing TKA implants of more suitable sizes and aspect ratios for Indian patients. Larger and more collaborative studies with other Asian ethnic researchers to reach a consensus are necessary to push for industrial manufacturing of size-appropriate implants for better long-term outcomes.
